# N-Terminal Lipid Modification Is Required for the Stable Accumulation of CyanoQ in *Synechocystis* sp. PCC 6803

**DOI:** 10.1371/journal.pone.0163646

**Published:** 2016-09-22

**Authors:** Andrea D. Juneau, Laurie K. Frankel, Terry M. Bricker, Johnna L. Roose

**Affiliations:** Department of Biological Sciences, Biochemistry and Molecular Biology Section, Louisiana State University, Baton Rouge, Louisiana, 70803, United States of America; University of California—Davis, UNITED STATES

## Abstract

The CyanoQ protein has been demonstrated to be a component of cyanobacterial Photosystem II (PS II), but there exist a number of outstanding questions concerning its physical association with the complex. CyanoQ is a lipoprotein; upon cleavage of its transit peptide by Signal Peptidase II, which targets delivery of the mature protein to the thylakoid lumenal space, the N-terminal cysteinyl residue is lipid-modified. This modification appears to tether this otherwise soluble component to the thylakoid membrane. To probe the functional significance of the lipid anchor, mutants of the CyanoQ protein have been generated in *Synechocystis* sp. PCC 6803 to eliminate the N-terminal cysteinyl residue, preventing lipid modification. Substitution of the N-terminal cysteinyl residue with serine (Q-C22S) resulted in a decrease in the amount of detectable CyanoQ protein to 17% that of the wild-type protein. Moreover, the physical properties of the accumulated Q-C22S protein were consistent with altered processing of the CyanoQ precursor. The Q-C22S protein was shifted to a higher apparent molecular mass and partitioned in the hydrophobic phase in TX-114 phase-partitioning experiments. These results suggest that the hydrophobic N-terminal 22 amino acids were not properly cleaved by a signal peptidase. Substitution of the entire CyanoQ transit peptide with the transit peptide of the soluble lumenal protein PsbO yielded the Q-SS mutant and resulted in no detectable accumulation of the modified CyanoQ protein. Finally, the CyanoQ protein was present at normal amounts in the PS II mutant strains *ΔpsbB* and *ΔpsbO*, indicating that an association with PS II was not a prerequisite for stable CyanoQ accumulation. Together these results indicate that CyanoQ accumulation in *Synechocystis* sp. PCC 6803 depends on the presence of the N-terminal lipid anchor, but not on the association of CyanoQ with the PS II complex.

## Introduction

Photosystem II (PS II) is the light-driven water-plastoquinone oxidoreductase found in the thylakoid membranes of cyanobacteria, algae, and plants, splitting water into molecular oxygen and reducing plastoquinone to plastoquinol. This membrane protein complex contains at least seventeen intrinsic membrane protein subunits [1]. In addition to these core subunits, a number of extrinsic proteins are associated with the lumenal side of the enzyme [2]. The complement of extrinsic proteins differs between the green plant and cyanobacterial lineages. In green plants, the PsbO, PsbP and PsbQ subunits are present while cyanobacteria contain the PsbO, PsbU, PsbV, CyanoQ and, possibly the CyanoP, proteins. These extrinsic proteins associate with the lumenal face of PS II and collectively interact with each other and with the intrinsic components to support oxygen evolution. While the PsbO subunit is common to all oxygenic photosynthetic organisms, the cyanobacterial CyanoQ and CyanoP proteins are only distantly related to the higher plant extrinsic subunits PsbQ and PsbP, respectively. Functional studies indicate that they are not equivalent [2–4].

The CyanoQ protein, while having been shown unequivocally to be a component of cyanobacterial PS II [5], is not resolved in current PS II X-ray structures [1, 6] and is apparently lost during the crystallization process or is present in sub-stoichiometric amounts [7]. Consequently, little is known as to how this subunit interacts with the other extrinsic proteins present on the lumenal side of PS II (PsbO, PsbU and PsbV) or the intrinsic components of PS II. A recent study, using protein crosslinking coupled with tandem mass spectrometry, mapped the CyanoQ protein to a location within PS II close to both the CP47 and PsbO subunits at the PS II dimer interface [8]. This study also demonstrated that loss of the PsbO subunit resulted in the absence of CyanoQ in PS II complexes.

Reverse genetic studies of these proteins have provided insights into their functions, but have provided only an incomplete picture of their interactions. While the *ΔcyanoQ* mutant alone exhibits a very subtle PS II phenotype, the absence of CyanoQ in combination with the deletion of other extrinsic subunits results in severe photosynthetic defects [9–11]. For example, the *ΔcyanoQΔpsbV* double mutant cannot grow photoautotrophically in standard medium and has a significantly reduced PS II quantum yield [10].

Early studies of CyanoQ-containing PS II complexes suggested that the protein was one of the last extrinsic subunits to associate with the complex [5]. PS II complexes containing the CyanoQ protein were the most stable and exhibited the highest activity for oxygen evolution. The position of the CyanoQ protein at the dimer interface, based on the cross-linking data, was also consistent with CyanoQ being one of the last PS II components to assemble into the complex [8]. Interestingly, further analysis of a second population of CyanoQ-containing PS II complexes suggested an additional role for the CyanoQ protein in the photosystem [12]. In this study, a PS II complex containing the PsbO protein and four copies of the CyanoQ protein, but lacking the PsbU and PsbV subunits, was characterized. These results suggested that CyanoQ could interact with the core PS II subunits much earlier in the assembly pathway, possibly indicating additional roles for this component in the assembly process.

One distinctive feature of the CyanoQ protein is that, unlike the extrinsic proteins PsbO, PsbU and PsbV, CyanoQ is a lipoprotein (as is CyanoP) [11, 13, 14]. The extrinsic components, PsbO, PsbU and PsbV, have signal peptides at their N-termini targeting these subunits to the lumenal space. The signal peptides are cleaved by Signal Peptidase I, yielding soluble mature proteins in the thylakoid lumen. In contrast, the CyanoQ transit peptide contains a lipobox [15] that directs processing by Signal Peptidase II [16]. During maturation the targeted cysteinyl residue (proCyanoQ:^22^C) is initially covalently modified yielding an S-diacylglycerol-^22^C. Signal Peptidase II then cleaves proCyanoQ between amino acids 21 and 22, yielding an N-terminal S-diacylglycerol-modified cysteinyl residue. Subsequently, the N-terminus is acylated. Because of these lipid modifications the mature CyanoQ protein is tethered to the thylakoid membrane as a monotropic membrane protein [11, 14]. The precise identity of the lipid modifications is unclear. When overexpressed in *E*. *coli*, mass spectrometric analysis indicated that CyanoQ contains both the N-acyl and S-diacylglycerol modifications. The principal fatty acids present included two palmitates and one palmitoleate (low intensity signals for 9,10-methylene-hexadecanoate and vaccenate were also observed) [13]. It is unknown if native CyanoQ contains this same cohort of lipid modifications. The lipid modification of the CyanoQ protein is a key physical difference in comparison to its higher plant counterpart, PsbQ. Neither PsbQ nor any of the higher plant extrinsic proteins are tethered to the thylakoid membrane by a lipid anchor, and all of these can easily be washed away from the membrane by high-salt treatment (i.e. 1 M CaCl_2_).

A second important difference between CyanoQ and PsbQ is its mode of transport into the lumenal compartment. In higher plants, both PsbQ and PsbP are transported via the *Δ*pH-requiring TAT pathway and their transit peptide sequences contain the canonical twin arginine motif [17] that is uniformly conserved in chloroplasts, mitochondria, Gram-negative and -positive bacteria, and the Archaea. Higher plant PsbO is transported by the ATP-requiring Sec pathway [18]. Interestingly, neither proCyanoQ nor proCyanoP contain this twin arginine motif and both are predicted to be transported into the lumen by the Sec pathway (as is cyanobacterial PsbO). Additionally, while both precursor proteins contain a lipobox, neither possesses the aspartyl residue at position +2 of the mature protein. This aspartyl residue is required for lipobox proteins to avoid transport to the outer membrane [16, 19]. In *E*. *coli*, proteins lacking this aspartyl residue are translocated to the outer membrane using the LolABCDE transport proteins. Interestingly, *Synechocystis* sp. PCC 6803 (henceforth, *Synechocystis*) lacks recognizable homologues for many of these components. Finally, the PsbQ protein in higher plants is one of five evolutionarily related proteins. In Arabidopsis, two of these variants are PsbQ proteins that interact with PS II, while the other thee are termed PsbQ-Like (PQL) homologs which function within the NADPH-Dehydrogenase Complex [3]. With these numerous differences between the PsbQ and CyanoQ proteins, it has been difficult to ascertain the specific role of CyanoQ and its lipid modification. Moreover, it is difficult to draw clear functional parallels for the protein processing and translocation machinery in cyanobacteria from either Gram-negative bacteria or plants. Therefore, assumptions about these processes and the functional consequences for proteins may not be accurate.

To probe the functional significance of the lipid anchor, two different *cyanoQ* mutants have been generated in the cyanobacterium *Synechocystis* which eliminate this feature from the mature CyanoQ protein. In the mutant Q-C22S a serine residue replaced the cysteine residue at position 22, eliminating the N-terminal cysteinyl residue and consequently the lipid modification. In the second strain, Q-SS, the sequence corresponding to the signal peptide from the cyanobacterial *psbO* gene replaced that of the *cyanoQ* gene. Here we characterize the Q-C22S and Q-SS strains with respect to the accumulation and physical properties of their altered CyanoQ proteins. Additionally, we have examined the ability of wild-type CyanoQ to accumulate in the thylakoid membrane in the absence of its association with PS II. Our results indicate that the N-terminal lipid modification in CyanoQ is required for accumulation of this subunit of PS II.

## Materials and Methods

### Strains and growth conditions

The wild-type *Synechocystis* strain used in this study was the glucose-tolerant variety described by Williams in 1988 [20]. The wild-type, Q-Ctrl, Q-C22S, Q-SS, *ΔpsbB*, and *ΔpsbO* strains of *Synechocystis* were grown in BG-11 medium [21] supplemented with 5 mM glucose and 10 μM DCMU at 30°C under 30 μmole photons m^-2^ s^-1^. The Q-Ctrl and Q-C22S strains were supplemented with 10 μg/ml kanamycin; the Q-SS strain was supplemented with 5 μg/ml gentamycin. The *ΔpsbB* and *ΔpsbO* strains were supplemented with 10 μg/ml spectinomycin and the construction of these strains has been described previously [22, 23] The Q-Ctrl, Q-C22S and Q-SS strains were constructed as described below.

### Mutant construction

The wild-type *Synechocystis cyanoQ* gene including 200 bp of upstream flanking DNA was cloned into a pUC18-based plasmid at the NdeI and XbaI sites using the cyanoQF and cyanoQR primers (refer to Table 1 for all primer sequences). A kanamycin-resistance marker from the pUC4K plasmid [24] was cloned into the KpnI site using the KanF and KanR primers. DNA corresponding to the 500 bp immediately downstream of the *cyanoQ* locus was cloned into the SacI and EcoRI sites with the cyanoQDSF and cyanoQDSR primers. The resulting WTcyanoQ plasmid was used to transform a glucose-tolerant wild-type *Synechocystis* strain to yield the kanamycin-resistant Q-Ctrl strain.

**Table 1:** Primer Sequences.

Primer Name	Sequence
CyanoQF	GGAATTCCATATGGCTGCGGCCAACATTAAGCG
CyanoQR	CTAGTCTAGACTAGCTAGCTTGGGGCAACAGG
KanF	GGGGTACCGATCCGTCGACCTGCAGGG
KanR	GGGGATCCGGGGGAAAGCCACGTTGTG
CyanoQDSF	GTCCGAGCTCGCATCCTTGAGCAAGATCA
CyanoQDSR	CGGTCCTCGTTAGTAGCAGTAGCCCCCAG
CyanoQC22SF	CGGTCCTCGTTAGTAGCAGTAGCCCCCAG
CyanoQC22SR	CTGGGGGCTACTGCTACTAACGAGGACCG
PsbOSignalF	ACATGCATGCATGAGGTTTCGTCCGTCTATTG
PsbOSignalR	GACGTCGACCGCAAAGGCACTGCCGCT
UScyanoQF	GGAATTCCATATGACTAGCCAAAGCGGCGATCG
UScyanoQR	ACATGCATGCGGGTTACGGTGTTATTGACATTG
MatcyanoQF	GCGTCGACAGTAGCCCCCAGGTGGAAATCC
MatcyanoQR	gcGGATCCTAGCTAGCTTGGGGCAACAGG
CyanoQsegF	TTGCAGGTCTACGTCAATCCGA
CyanoQsegR	AAATCTTTCCCGCCGAAGCACT

To generate the Q-C22S point mutant, in which residue ^22^C was substituted with ^22^S, site-directed mutagenesis of the WTcyanoQ plasmid was performed using the cyanoQC22SF and cyanoQC22SR primers according to the QuikChange II Site-Directed Mutagenesis Kit (Agilent). The resulting Q-C22S plasmid was used to transform a glucose-tolerant wild-type *Synechocystis* strain to yield the kanamycin-resistant Q-C22S strain. The mutation was confirmed in both the plasmid and the genomic DNA of the *Synechocystis* Q-C22S strain by DNA sequencing.

For the Q-SS mutant, the N-terminal signal peptide of the *cyanoQ* gene (residues 1–22) was replaced with the N-terminal signal peptide of the *psbO* gene. To generate this mutant, the portion of the *psbO* gene corresponding to amino acid residues 1–30 was amplified with primers PsbOSignalF and PsbOSignalR and cloned into the SphI and SalI sites of the pUC18 plasmid to yield the PsbOSignal/pUC18 plasmid. DNA corresponding to the sequence just upstream of the *cyanoQ* gene was amplified with the primers UScyanoQF and UScyanoQR to incorporate NdeI and SphI sites, respectively, and this PCR product was cloned into the PsbOsignal/pUC18 plasmid to yield the UScyanoQPsbOSignal/pUC18 plasmid. The DNA sequence corresponding to the mature CyanoQ protein coding region was amplified with the primers MatcyanoQF and MatcyanoQR, which incorporated SalI and BamHI restriction sites, respectively. The MatcyanoQ PCR fragment was cloned into the UScyanoQPsbOSignal/pUC18 plasmid at the SalI and BamHI sites to yield the UScyanoQPsbOSignalMatcyanoQ/pUC18 plasmid. The DNA sequence corresponding to 500 bp downstream of the *cyanoQ* gene was amplified with the cyanoQDSF and cyanoQDSR primers which incorporated SacI and EcoRI restriction sites, respectively. The cyanoQDS fragment was cloned into a pUC18 vector to yield the cyanoQDS/pUC18 plasmid. A DNA fragment conferring gentamycin resistance was cloned into the KpnI site of the cyanoQDS/pUC18 plasmid to yield the GmRcyanoQDS/pUC18 plasmid. The BamHI/EcoRI fragment of the GmRcyanoQDS/pUC18 plasmid containing the gentamycin-resistance gene and downstream *cyanoQ* DNA was cloned into the BamHI and EcoRI sites of the UScyanoQPsbOSignalMatcyanoQ/pUC18 plasmid to yield the Q-SS/pUC18 plasmid. This plasmid contained a chimeric gene with the *psbO* signal sequence fused to the sequence encoding the mature *cyanoQ* with an adjacent gentamycin-resistance gene at the 3’end and flanked on either side by sequence at the *cyanoQ* locus to ensure efficient double homologous recombination at the *cyanoQ* locus. The resulting Q-SS/PUC18 plasmid was used to transform a glucose-tolerant wild-type *Synechocystis* strain to yield the gentamycin-resistant Q-SS strain. Sequencing analysis of the *cyanoQ* locus confirmed that the chimeric gene was integrated into the genome as intended without additional mutations. Complete segregation of all strains used in this study was confirmed by PCR analysis using the cyanoQsegF and cyanoQsegR primers.

### Electrophoresis and Protein Detection

Thylakoid membranes were isolated as described in Zhang et al. [25]. Chlorophyll (chl) concentrations were measured by the method of Lichtenthaler [26]. Samples containing 5 μg chl or an equivalent volume (for the soluble, non-chl-containing fractions) were loaded per lane. For soluble protein fractions, cells were broken as described above and the resulting supernatant was saved for analysis. Soluble protein samples were quantified by BCA Assay (Bio-Rad) using a standard curve of bovine serum albumin. LDS-PAGE was performed under conditions described by Delepelaire and Chua [27] in gradient 12.5–20% polyacrylamide gels. The resolved proteins were electroblotted onto PVDF membranes (Immobilon-P, Millipore Corp.). After blocking for 2 h with 5% nonfat dry milk in 150 mM NaCl, 10 mM Tris-HCl, pH 7.4, the blots were washed extensively with 150 mM NaCl, 10 mM Tris-HCl, pH 7.4. The blots were then incubated with diluted primary antibody followed by incubation with an anti-rabbit or anti-mouse IgG-peroxidase conjugate (Sigma). The labeled protein was detected using chemiluminescence (Pierce). For semi-quantitative analysis of protein abundance, a standard curve for the chemiluminescence signal for CyanoQ was produced using a dilution series of Q-Ctrl thylakoids. The chemilumenescent signals from the mutated CyanoQ proteins were compared to this standard curve and analyzed using NIH ImageJ software [28].

### TX-114 Phase Partitioning

Triton X-114 phase partitioning of thylakoid membranes was performed essentially as previously described [29, 30] at 100 μg chl/mL. Briefly, the thylakoid membranes which were suspended in 50 mM MES-NaOH, pH 6.0, 10 mM MgCl_2_, 5 mM CaCl_2_, 25% glycerol were brought to 1% TX-114 using a 20% stock TX-114 solution. The membranes were incubated on ice for 5 min and then incubated at 37°C for 5 min. During this second incubation the clear detergent solution became cloudy due to condensation of TX-114 micelles. After centrifugation at 14000 × *g* for 5 min at room temperature, the solution exhibited phase separation with a dark green, detergent-enriched lower phase (containing the intrinsic and monotropic membrane proteins) and a pale green, detergent-depleted upper phase (containing extrinsic membrane and soluble proteins). The upper and lower phases were carefully collected. The lower phase was brought to ≈100 μg chl/mL by the addition of 50 mM MES-NaOH, pH 6.0, 10 mM MgCl_2_, 5 mM CaCl_2_, 25% glycerol and the upper phase was brought to 1% TX-114. Phase partitioning was then repeated for both phases.

## Results and Discussion

### Strain Construction

**Fig 1:**
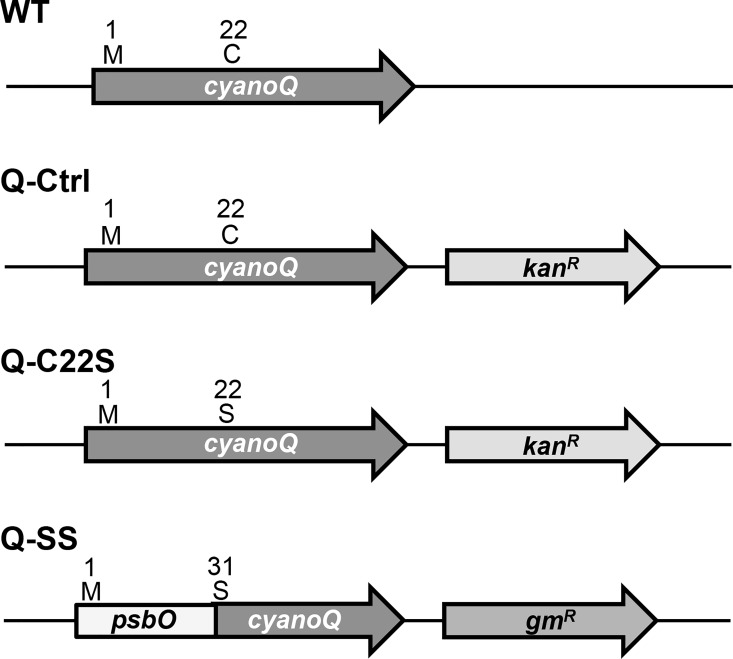
*CyanoQ* Gene Structure and Mutant Constructs.

The lumenal proteins of cyanobacteria are targeted to their subcellular location by signal peptides at their N-termini. In the case of the PS II extrinsic proteins PsbO, PsbU and PsbV, the signal peptides are recognized by Signal Peptidase I upon transport across the thylakoid membrane. Proteolytic cleavage of these signal peptides yields soluble proteins in the lumenal space. In contrast, the extrinsic lipoproteins CyanoQ, CyanoP, and Psb27 have signal peptides like those recognized by Signal Peptidase II and upon targeting across the thylakoid membrane and signal sequence cleavage, the resulting N-terminal cysteinyl residue is covalently modified with a lipid moiety. In order to address the functional significance of the N-terminal lipid modification of the CyanoQ protein, strains of *Synechocystis* which eliminated the modified cysteinyl residue were constructed (Fig 1). In the Q-C22S strain, the ^22^C was mutated to ^22^S, and for selection purposes a kanamycin-resistance marker gene was engineered just downstream of the *cyanoQ* gene. In the Q-SS strain, the N-terminal signal sequence of PsbO (residues 1–30) was fused with the sequence corresponding to the coding region of the mature CyanoQ protein (residues 31–157). The mature portion of the CyanoQ protein in the Q-SS chimeric sequence corresponds to residues 23–149 of the wild-type CyanoQ protein. The cysteine residue corresponding to residue 22 in the wild-type CyanoQ protein was not included in the Q-SS chimeric sequence. In the case of the Q-SS strain, a gentamycin-resistance marker gene was engineered just downstream of the *cyanoQ* locus for selection purposes. A control strain (Q-Ctrl) was also constructed which contained the wild-type *cyanoQ* gene with the downstream kanamycin-resistance gene.

The LipoP program predicts lipoproteins and their Signal Peptidase II cleavage sites within their amino acid sequences [31]. Analysis of the wild-type CyanoQ protein sequence predicted a Signal Peptidase II cleavage site between residues 21 and 22 (LVS-CSS). This is in agreement with previously published reports that demonstrated that ^22^C is the N-terminal residue of the mature protein and modified with lipid moieties [13]. Analysis of the Q-C22S sequence showed that the Signal Peptidase II cleavage site had been eliminated, but a Signal Peptidase I cleavage site was predicted between residues 25 and 26. The SignalP program was used to predict the location of a signal peptide cleavage site for the Q-C22S sequence [32]. This program predicted a cleavage between residues 22 and 23 (LVSS-SS). Consequently, an alternative cleavage site exists on the Q-C22S protein, but the lipid modification site is no longer present. For the Q-SS amino acid sequence, the signal peptide cleavage position was analogous to that predicted for the PsbO amino acid sequence between residues 28 and 29 (AFA-VD) and the N-terminal residue of the mature protein would be ^29^V with the lipid-modification site, consequently, being eliminated. The cysteine residue corresponding to ^22^C in the wild-type CyanoQ protein was not included in the Q-SS construct.

### Accumulation of the CyanoQ Proteins

**Fig 2:**
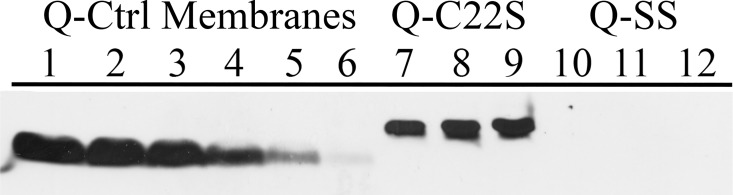
Detection of CyanoQ in Membrane Samples. Membranes from Q-Ctrl were used as a standard series for CyanoQ detection. Lane 1, 5 μg Chl; Lane 2, 2.5 μg Chl; Lane 3, 1.25 μg Chl; Lane 4, 0.5 μg Chl; Lane 5 0.25 μg Chl, Lane 6, 0.05 μg Chl. Membranes from Q-C22S (Lane 7, 5 μg Chl; Lane 8, 10 μg Chl; Lane 9, 12.5 μg Chl) and Q-SS (Lane 10, 5 μg Chl; Lane 11, 10 μg Chl; Lane 12, 12.5 μg Chl). After immunoblotting all lanes were probed with an anti-CyanoQ protein antibody and detected by chemiluminescence.

In order to characterize the mutant CyanoQ proteins lacking the N-terminal lipid modification, membrane and soluble protein samples from the Q-Ctrl, Q-C22S and Q-SS strains were analyzed using LDS-PAGE. The accumulation of the CyanoQ protein in the thylakoid membranes of the Q-C22S and Q-SS strains is shown in Fig 2. There was reduced accumulation of the CyanoQ protein in the Q-C22S strain. Comparison with a series of known amounts of Q-Ctrl thylakoid membranes indicated that the CyanoQ protein accumulated to only 17% that of control membranes when analyzed on a chl basis. The CyanoQ protein in the Q-C22S strain was also shifted to a slightly higher apparent molecular mass than wild-type CyanoQ in the Q-Ctrl strain. No CyanoQ could be detected in the Q-SS mutant.

**Fig 3:**
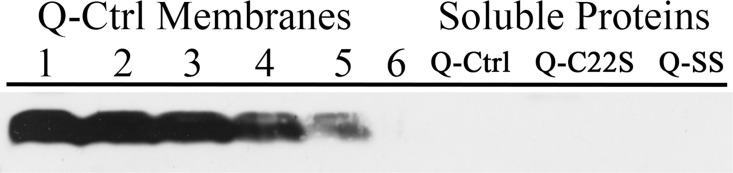
CyanoQ Detection in Soluble Fractions. Membranes from Q-Ctrl were used as a standard series for CyanoQ detection. Lane 1, 5 μg Chl; Lane 2, 2.5 μg Chl; Lane 3, 1.25 μg Chl; Lane 4, 0.5 μg Chl; Lane 5 0.25 μg Chl, Lane 6, 0.05 μg Chl. Soluble fractions from Q-Ctrl, Q-C22S and Q-SS contain 100 μg of protein. After immunoblotting all lanes were probed with an anti-CyanoQ protein antibody and detected by chemiluminescence.

Because the mutations introduced in the Q-C22S and Q-SS strains could hypothetically yield a soluble form of the CyanoQ protein, which might no longer associate with the thylakoid membranes, soluble fractions from the Q-Ctrl, Q-C22S and Q-SS strains also were analyzed for the presence of CyanoQ (Fig 3). The CyanoQ protein was not detected in the soluble fractions of any of the strains analyzed. Based on these results, we conclude that the absence of the N-terminal lipid modification significantly affects the accumulation of the CyanoQ protein in both the Q-C22S and Q-SS mutants.

### Phase-Partitioning Properties of the Q-C22S Protein

**Fig 4:**
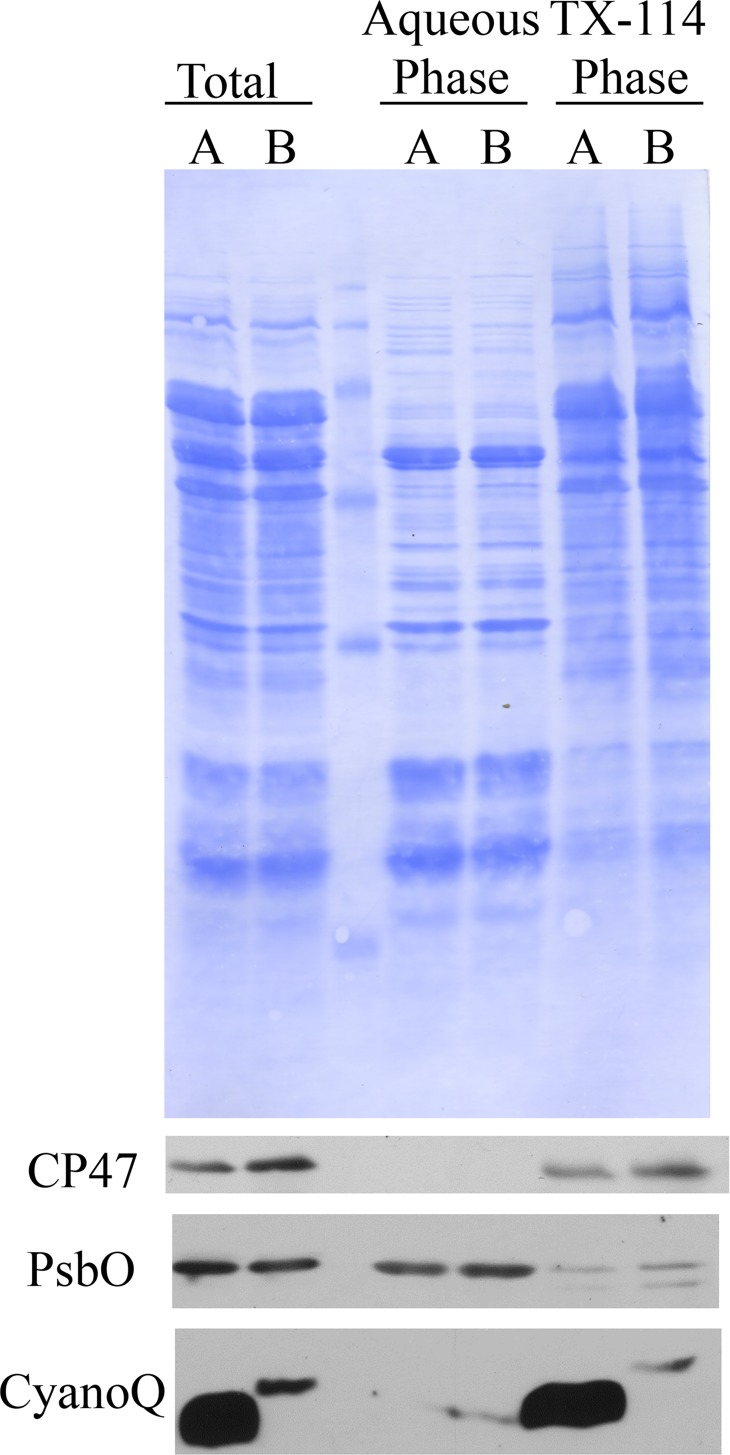
TX-114 Phase Partitioning. Thylakoid membranes from Q-Ctrl (A) and Q-C22S (B) were partitioned into an aqueous fraction and a TX-114 detergent fraction. The total membranes along with the aqueous and TX-114 phases were separated by LDS-PAGE. Proteins were detected using Coomassie blue. After immunoblotting, specific antibodies against the CP47, PsbO and CyanoQ proteins were used to detect these components by chemiluminescence.

Because there was some accumulation of the CyanoQ protein in the Q-C22S strain, TX-114 phase partitioning was used to characterize its phase-partitioning properties relative to that of the wild-type CyanoQ protein (Fig 4). This technique differentially partitions soluble extrinsic membrane proteins into an aqueous phase, and lipophilic intrinsic and monotropic proteins into a TX-114 phase. As expected, the transmembrane protein CP47 partitioned in the TX-114 phase for the Q-Ctrl and Q-C22S strains, while the soluble lumenal protein PsbO partitioned in the aqueous phases for these strains. Because of its N-terminal lipid modification the otherwise hydrophilic CyanoQ protein partitioned in the TX-114 phase for the Q-Ctrl strain. This is consistent with previous results for the wild-type CyanoQ protein [11] and indicates that this subunit is a monotropic membrane protein tethered to the lumenal face of the thylakoid membrane by its N-terminal lipid anchor. Interestingly, the CyanoQ protein in the Q-C22S strain also partitioned in the TX-114 phase, suggesting that the small amount of modified protein which did accumulate in the mutant exhibited hydrophobic characteristics. Given the shift to a higher apparent molecular mass of CyanoQ in the Q-C22S strain, we hypothesize that the signal sequence (amino acids 1–22) of the precursor Q-C22S CyanoQ protein is not properly cleaved to form a mature Q-C22S CyanoQ. Retention of this predominantly hydrophobic signal sequence in the Q-C22S CyanoQ protein would account for the TX-114 phase partitioning result.

### Accumulation of Wild-Type CyanoQ in Other Mutant Backgrounds

**Fig 5:**
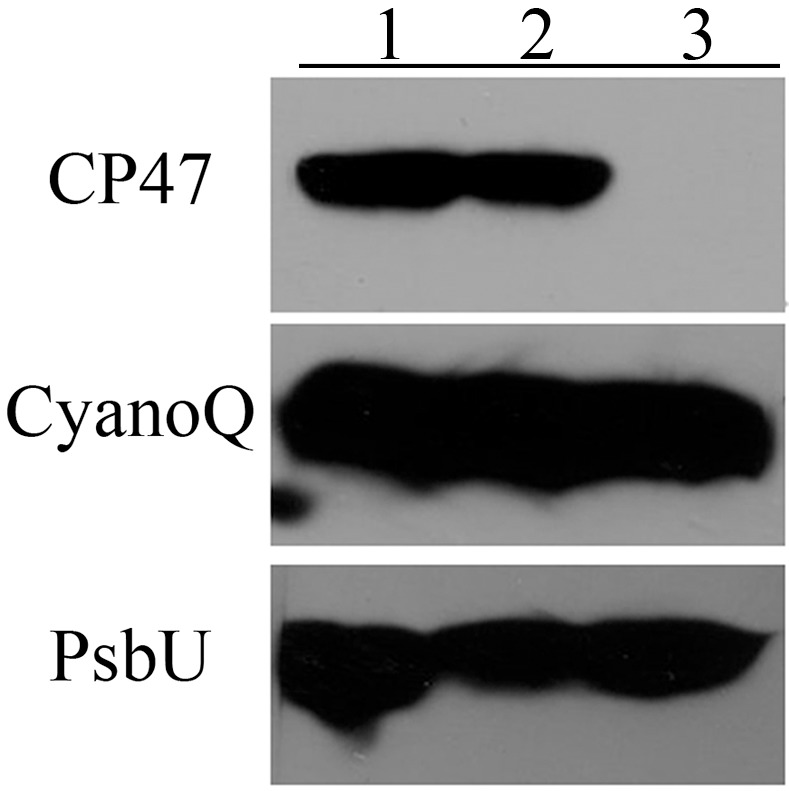
PS II Protein Accumulation in other Genetic Backgrounds. Membranes from Lane 1, wild type; Lane 2, *ΔpsbO*; and, Lane 3, *ΔpsbB*, corresponding to 5 μg Chl per lane. After immunoblotting, specific antibodies against the CP47, CyanoQ and PsbU proteins were used to detect these components by chemiluminescence.

It is possible that the loss of the N-terminal lipid modification of CyanoQ prevents the protein from associating with PS II, and that this lack of association with the photosystem leads to its rapid turnover and a failure to accumulate in the thylakoid membranes. To investigate this possibility we examined the accumulation of wild-type CyanoQ protein in genetic backgrounds which would preclude its association with PS II. Two different genetic backgrounds were investigated. In the *ΔpsbB* strain, the CP47 protein is absent and PS II complexes do not accumulate [22]. In the *ΔpsbO* strain, the PsbO protein is absent and, while PS II complexes assemble, CyanoQ cannot associate with the photosystem [8]. Consequently, CyanoQ cannot associate with PS II complexes in either of these strains. If the association of CyanoQ with PS II modulates its stability, CyanoQ should be unstable in both genetic backgrounds. Fig 5 shows the accumulation of the CyanoQ protein, along with the membrane protein CP47 and the soluble extrinsic protein PsbU in both the *ΔpsbB* and *ΔpsbO* backgrounds. The CP47 protein is present in both the wild-type strain and the *ΔpsbO* strain, but is clearly absent in the *ΔpsbB* strain. The soluble extrinsic protein PsbU also accumulates in all three strains. A similar result was observed in the *Synechocystis ΔctpA* mutant. In this strain PsbO, PsbU and PsbV accumulate but do not associate with the photosystem [33]. Additionally, in a *Synechocystis Δpsb*A mutant, which lacks assembled PS II complexes, PsbO also accumulates [34]. In higher plants it has been demonstrated that a stable pool of extrinsic proteins can exist in the thylakoid lumen independent of PS II [35]. Apparently, this finding also applies to *Synechocystis*. Interestingly, our results indicate that the CyanoQ protein also accumulates in the *ΔpsbB* and *ΔpsbO* strains, indicating that the wild-type CyanoQ protein, containing its normal N-terminal lipid modification, can stably accumulate within the thylakoid membrane independent of the accumulation of PS II or the presence of PsbO. Consequently, it appears that the lack of accumulation of CyanoQ in the Q-C22S and Q-SS mutants, in which the normal N-terminal lipid anchor absent, is not due to any inherent problems with its association with PS II in these strains.

## Conclusion

In *Synechocystis*, three lipoproteins are associated with PS II, CyanoQ, CyanoP and Psb27 [14]. While each of proteins has a homolog in higher plants, these components do not contain lipid modifications. This key physical difference has important implications for their function and association with PS II in the prokaryotic and eukaryotic systems. The presence of the lipid modification has also presented certain technical limitations to experiments which can be performed elucidating their function and structural association with cyanobacterial PS II. For example, these proteins cannot be removed from the thylakoid membrane or from detergent particle preparations by a salt-wash treatment, so reconstitution experiments, which have been extremely useful in higher plant systems, are impossible. Additionally, the procedures for obtaining high quality crystals for structural studies appear to result in the loss of these lipoproteins from the photosystem, and consequently, no cyanobacterial structures containing these components are available.

In this communication we have examined the functional significance of the N-terminal lipid modification of the CyanoQ protein. This protein was selected because it is easily detected and has been previously shown to be associated with a large proportion of PS II complexes. Our results indicate that the lipid modification of the CyanoQ N-terminus is required for its stable accumulation in *Synechocystis*. Site-directed modification of the targeted cysteinyl residue (^22^C) to a seryl residue in the Q-C22S mutant allows limited accumulation of a modified mutated protein. The higher apparent molecular mass of the modified protein is consistent with improper signal sequence processing and the presence of an unprocessed signal sequence. The largely hydrophobic transit sequence appears to confer hydrophobic characteristics to the Q-C22S protein, anchoring it to the thylakoid membrane. Unlike higher plant PsbQ and PsbP, CyanoQ (and CyanoP) lacks the twin-arginine motif recognized by the TAT translocation machinery. Consequently, CyanoQ is translocated by the Sec pathway, as is cyanobacterial PsbO. Our observation that the Q-SS mutant does not accumulate the Q-SS CyanoQ protein in either membrane-bound or soluble forms indicates that a Sec transit sequence, alone, is insufficient to allow accumulation of the protein. Both the lipobox and the target cysteinyl residue must be present.

## References

[1] Umena Y, Kawakami K, Shen J-R, Kamiya N. Crystal structure of oxygen-evolving Photosystem II at a resolution of 1.9 Å. Nature. 2011;473:55–60. 10.1038/nature09913 21499260

[2] Bricker TM, Roose JL, Fagerlund RD, Frankel LK, Eaton–Rye JJ. The extrinsic proteins of Photosystem II. Biochim Biophys Acta. 2012;1817:121–42. 10.1016/j.bbabio.2011.07.006 21801710

[3] Ifuku K. The PsbP and PsbQ family proteins in the photosynthetic machinery of chloroplasts. Plant Physiol Biochem. 2014;81:108–14. 10.1016/j.plaphy.2014.01.001 24477118

[4] Roose JL, Frankel LK, Mummadisetti MP, Bricker TM. The extrinsic proteins of Photosystem II: Update. Planta. 2016;243:889–908. 10.1007/s00425-015-2462-6 26759350

[5] Roose JL, Kashino Y, Pakrasi HB. The PsbQ protein defines cyanobacterial Photosystem II complexes with highest activity and stability. Proc Natl Acad Sci (USA). 2007;104:2548–53. 17287351 10.1073/pnas.0609337104 PMC1892988

[6] Suga M, Akita F, Hirata K, Ueno G, Murakami H, Nakajima Y, et al Native structure of Photosystem II at 1.95 angstrom resolution viewed by femtosecond X-ray pulses. Nature. 2015;517:99–103. 10.1038/nature13991 25470056

[7] Michoux F, Boehm M, Bialek W, Takasaka K, Maghlaoui K, Barber J, et al Crystal structure of CyanoQ from the thermophilic cyanobacterium *Thermosynechococcus elongatus* and detection in isolated Photosystem II complexes. Photosyn Res. 2014;122:57–67. 10.1007/s11120-014-0010-z 24838684 PMC4180030

[8] Liu H, Zhang H, Weisz DA, Vidavsky I, Gross ML, Pakrasi HB. MS-based cross-linking analysis reveals the location of the PsbQ protein in cyanobacterial Photosystem II. Proc Natl Acad Sci (USA). 2014;111:4638–43. 24550459 10.1073/pnas.1323063111 PMC3970497

[9] Thornton L, Ohkawa H, Roose J, Kashino Y, Keren N, Pakrasi H. Homologs of plant PsbP and PsbQ proteins are necessary for regulation of Photosystem II activity in the cyanobacterium *Synechocystis* 6803. Plant Cell. 2004;16:2164–75. 15258264 10.1105/tpc.104.023515 PMC519205

[10] Summerfield TC, Shand JA, Bentley FK, Eaton-Rye JJ. PsbQ (sll1638) in *Synechocystis* sp. PCC 6803 is required for Photosystem II activity in specific mutants and in nutrient-limiting conditions. Biochemistry. 2005;44:805–15. 15641809 10.1021/bi048394k

[11] Kashino Y, Inoue-Kashino N, Roose J, Pakrasi H. Absence of the PsbQ protein results in destabilization of the PsbV protein and decreased oxygen evolution activity in cyanobacterial Photosystem II. J Biol Chem. 2006;281:20834–41. 16723351 10.1074/jbc.M603188200

[12] Liu H, Weisz DA, Pakrasi HB. Multiple copies of the PsbQ protein in a cyanobacterial photosystem II assembly intermediate complex. Photosynthesis research. 2015;126(2–3):375–83. Epub 2015/03/25. 10.1007/s11120-015-0123-z . 25800517

[13] Ujihara T, Sakurai I, Mizusawa N, Wada H. A method for analyzing lipid-modified proteins with mass spectrometry. Anal Biochem. 2008;374:429–31. 18078799 10.1016/j.ab.2007.11.014

[14] Fagerlund RD, Eaton-Rye JJ. The lipoproteins of cyanobacterial Photosystem II. J Photochem Photobiol B: Biol. 2011;104:191–203. 10.1016/j.jphotobiol.2011.01.022 21349737

[15] Hayashi S, Wu HC. Lipoproteins in bacteria. J Bioenerg Biomembr. 1990;22:451–71. 2202727 10.1007/BF00763177

[16] Tokuda H, Matsuyama S-i. Sorting of lipoproteins to the outer membrane in *E*. *coli*. Biochim Biophys Acta. 2004;1693:5–13. 15276320 10.1016/j.bbamcr.2004.02.005

[17] Lee PA, Tullman-Ercek D, Georgiou G. The bacterial twin-arginine translocation pathway. Ann Rev Microbiol. 2006;60:373–95. 16756481 10.1146/annurev.micro.60.080805.142212 PMC2654714

[18] Mori J, Cline K. Post-translational protein translocation into thylakoids by the Sec and ΔpH-dependent pathways. Biochim Biophys Acta. 2001;1541:80–90. 11750664 10.1016/s0167-4889(01)00150-1

[19] Okuda S, Tokuda H. Lipoprotein sorting in bacteria. Ann Rev Microbiol. 2011;65:239–59. 21663440 10.1146/annurev-micro-090110-102859

[20] Williams JGK. [85] Construction of specific mutations in photosystem II photosynthetic reaction center by genetic engineering methods in Synechocystis 6803 Methods in Enzymology. Volume 167: Academic Press; 1988 p. 766–78.

[21] Rippka R, J., Deruelles JB, Waterbury M, Herdman M, Stanier RY. Genetic assignments, strain histories and properties of pure cultures of cyanobacteria. J Gen Microbiol. 1979;111:1–61.

[22] Putnam-Evans C, Bricker TM. Site-directed mutagenesis of the CPa-1 protein of Photosystem II: Alteration of the basic residue pair 384,385R to 384,385G leads to a defect associated with the oxygen-evolving complex. Biochemistry. 1992;31:11482–8. 1445882 10.1021/bi00161a029

[23] Chandler LE, Bartsevich VV, Pakrasi HB. Regulation of manganese uptake in *Synechocystis* 6803 by RfrA, a member of a novel family of proteins containing a repeated five-residues domain. Biochemistry. 2003;42:5508–14. 12731893 10.1021/bi027113a

[24] Taylor LA, Rose RE. A correction in the nucleotide sequence of the Tn903 kanamycin resistance determinant in pUC4K. Nucleic Acid Res. 1988;16:7762. 10.1093/nar/16.1.358 PMC334642 3340535

[25] Zhang S, Frankel LK, Bricker TM. The Sll0606 protein is required for Photosystem II assembly/stability in the cyanobacteroum *Synechocystis* sp. PCC 6803. J Biol Chem. 2010;285:32047–54. 10.1074/jbc.M110.166983 20724474 PMC2952206

[26] Lichtenthaler HK. Chlorophylls and carotenoids: pigments of photosynthetic biomembranes. Meth Enzymol. 1987;148:350–82.

[27] Delepelaire P, Chua NH. Lithium dodecyl sulfate/polyacrylamide gel electrophoresis of thylakoid membranes at 4 degrees C: Characterizations of two additional chlorophyll *a*-protein complexes. Proc Natl Acad Sci U S A. 1979;76(1):111–5. 16592604 10.1073/pnas.76.1.111 PMC382886

[28] Schneider CA, Rasband WS, Eliceiri KW. NIH Image to ImageJ: 25 years of image analysis. Nat Methods. 2012;9:671–5. 22930834 10.1038/nmeth.2089 PMC5554542

[29] Bricker TM, Sherman LA. Triton X-114 phase fractionation of maize thylakoid membrane proteins in the investigation of thylakoid membrane topology. FEBS Lett. 1982;149:197–202.

[30] Bricker TM, Sherman LA. Triton X-114 phase fractionation analysis of the thylakoid membranes of *Anacystis nidulans* R2. Arch Biochem Biophys. 1984;235:204–11. 6093709 10.1016/0003-9861(84)90269-8

[31] Juncker AJ, Willenbrock H, von Heijne G, Nielsen H, Brunak S, Krogh A. Prediction of lipoprotein signal peptides in gram-negative bacteria. Protein Sci. 2003;12:1652–62. 12876315 10.1110/ps.0303703 PMC2323952

[32] Petersen TN, Brunak S, von Heijne G, Nielsen H. SignalP 4.0: discriminating signal peptides from transmembrane regions. Nat Meth. 2011;8:785–6. 10.1038/nmeth.1701 21959131

[33] Roose J, Pakrasi H. Evidence that D1 processing is required for manganese binding and extrinsic protein assembly into Photosystem II. J Biol Chem. 2004;279:45417–22. 15308630 10.1074/jbc.M408458200

[34] Nilsson F, Andersson B, Jansson C. Photosystem II characteristics of a constructed *Synechocystis* 6803 mutant lacking synthesis of the D1 polypeptide. Plant Mol Biol. 1990;14:1051–4. 2129398 10.1007/BF00019402

[35] Ettinger WF, Theg SM. Physiologically active chloroplasts contain pools of unassembled extrinsic proteins of the photosynthetic oxygen-evolving enzyme complex in the thylakoid lumen. J Cell Biol. 1991;115:321–8. 1918144 10.1083/jcb.115.2.321 PMC2289146

